# Virus-mediated autoimmunity in Multiple Sclerosis

**DOI:** 10.1186/1740-2557-3-1

**Published:** 2006-02-19

**Authors:** Nikolaos Grigoriadis, Georgios M Hadjigeorgiou

**Affiliations:** 1 B' Department of Neurology, Laboratory of Experimental Neurology and Neuroimmunology, AHEPA University Hospital, 1 Stilp Kyriakidi Street, Aristotle University of Thessaloniki, Thessaloniki, 54636 Thessaloniki, Greece; 2 Department of Neurology, Neurogenetics Unit, Medical School, University of Thessaly, 22 Papakyriazi Street, 41222 Larissa, Greece

## Abstract

Epidemiological data suggest the notion that in Multiple Sclerosis (MS) is an acquired autoimmune disease and the cause may be an environmental factor(s), probably infectious, in genetically susceptible individuals. Several cases of viral induced demyelinatimg encephalomyelitis in human beings and in experimental models as well as the presence of IgG oligoclonal bands in the cerebrospinal fluid indicate that the infectious factor may be viral. However, the absence of a specific virus identification in MS central nervous system may hardly support this notion. On the other hand, the partial response of patients with MS to immunosuppressive and immunomodulatory therapy support the evidence of an autoimmune etiology for MS. However, the autoimmune hypothesis shares the same criticism with the infectious one in that no autoantigen(s) specific to and causative for MS has ever been identified. Nevertheless, the absence of identifiable infectious agent, especially viral does not rule out its presence at a certain time – point and the concomitant long term triggering of an autoimmune cascade of events thereafter. Several concepts have emerged in an attempt to explain the autoimmune mechanisms and ongoing neurodegeneration in MS on the basis of the infectious – viral hypothesis.

## Background

Multiple sclerosis (MS) is widely believed to be an autoimmune disorder characterized by multifocal lesions of the CNS myelin and accumulating clinical signs due to axonal damage [1]. The aetiology of MS has been debated several times since the disease was first described. Myelin is damaged due to an immune attack consisted of several pathways and molecules, leading to impaired nerve function. Autoantibodies and autoreactive T cells activated against myelin antigens such as myelin basic protein (MBP), proteolipid protein (PLP), and myelin oligodendrocyte glycoprotein (MOG), have been detected in MS patients [2]. The majority of researchers consider MS as a CD4^+ ^T-helper 1 (Th1)-mediated inflammatory demyelinating disease [3,4]. Several data indicate this consideration, such as the cellular composition of brain and cerebrospinal fluid (CSF)-infiltrating cells and data from studies in a widely used animal model for MS, the Experimental Alergic Encephalomyelitis (EAE) [5]. In the EAE model, myelin components emulsified in complete Freund's adjuvant (CFA) and injected in susceptible animals lead to a CD4^+^-mediated autoimmune disease that shares clinical, immunological and pathological similarities with MS. CFA creates an artificial inflammatory milieu that does not reflect the natural environment in which self or mimic peptides would be normally encountered. EAE may also be induced passively by transferring anti-myelin activated T-cells to naive animals (transfer EAE), a finding that clearly indicates the autoimmune component of the disease.

Studies on EAE indicated that cytokines, chemokines and adhesion molecules induce the recruitment of leukocytes from periphery to CNS throughout a disrupted blood brain barrier (BBB) and a cascade of inflammatory events is established within the CNS. Eventually, axonal degeneration and loss is the hallmark in the disease process leading to a long-term disability [6]. Although EAE may not be the ideal animal model for the disease, the model itself in combination with several other experimental and clinical data indicate that MS is an autoimmune disease [7]. However, the major criticism of the autoimmune hypothesis is that autoantigen(s) specific to and causative for MS has never been identified. In addition, although inflammation is considered to be a primary feature of demyelianating plaques thus favouring the autoimmune component in this process, recent reports indicate that demyelination may precede inflammation [8] On the other hand, there are reports proposing that MS is not an autoimmune disease but a genetically determined disorder characterized by metabolically dependent neurodegeneration [9]. The latter may imply that immune reaction in MS may be a secondary one to the ongoing degeneration of axons and neurons. Nevertheless, while the autoimmune model may not explain every aspect of MS, it is difficult to ignore the considerable evidence that immunity plays a major role in MS. Another intriguing idea regarding the aetiology of MS may be that the immune response in MS could result from a chronic viral infection rather than autoimmunity in the usual sense [10-12].

The possible involvement of viruses in the aetiology of MS is a rather controversial issue. Based on immigration data, it has been suggested that environmental factor(s) may trigger MS before the age of adolescence, while the disease is clinically silent until years later. It is also apparent that there is a genetic susceptibility related at least to the HLA system [13-15] Among monozygotic twins there is a 70% disconcordance of MS suggesting that an exogenous factor causes the disease[14]. Evidently, these indications lead to the hypothesis that MS is a disease triggered by an environmental factor in genetically susceptible individuals during childhood [12,16-20]. Moreover, it has been speculated that the environmental factor in MS could be a virus [21-23] In addition, abnormal immune response to a variety of viruses in MS patients as well as analogy with animal models and other human diseases in which viruses can cause diseases with long incubation periods, relapse, and demyelination, further support the concept that viruses may be implicated in the MS aetiology.

Although to date no virus has been recognized as a causative factor of MS, the possibility that both autoimmunity and neurodegeneration in MS may coexist and commonly be explained following a viral infection is reviewed here.

## Experimental and clinical evidence for a virus-related aetiology of MS

### Animal models

Various viruses have been found to induce demyelination in laboratory animals following various infection protocols. The most studied experimental demyelination is infection of mice with Theiler's murine encephalomyelitis virus (TMEV) [24]. TMEV (a higly cytolytic picovarious) infection in mice serves as a model to explain infectious and parainfectious mechanisms underlying CNS demyelination. TMEV infection of oligodendrocytes is productive, resulting in cell lysis and liberation of more virions. By contrast, TMEV infection in macrophages is restricted, and results in apoptosis of macrophages. TMEV antigen is abundant in the cytoplasm of apoptotic macrophages. Small amounts of TMEV are liberated from persistently infected macrophages leading to infection of more macrophages as well as oligodendrocytes. A persistent CNS infection is established as virus spreads from macrophage to macrophage. Virus released from macrophages can infect and damage more oligodendrocytes, thus adding to immunopathological destruction of myelin [25].

Another animal model of virus-induced demyelination is the one established in BALB/c mice following infection with JHM virus (coronavirus). This strain infects predominantly oligodendrocytes, and the induced demyelination is not preceded by inflammation or any immune – related mechanism [26,27]. The infected oligodendrocytes contain intracisternal virions [28] and this model may be considered as a case of demyelination resulting simply from a direct virus induced cytopathology of oligodendrocytes.

Autoimmune responses to myelin antigens are observed following infection with TMEV. Despite the fact that this autoimmunity to myelin components may not play a major role in the initiation of demyelination, it may probably contribute to lesion progression in chronically diseased animals [29].

CNS demyelination in host animals may also occur following infection of other viruses such as in mice with JHM or MHV-4 (coronaviruses), dogs with canine distemper virus, and sheep and goats with Visna virus and caprine arthritis-encephalitis virus. Each of these viruses is capable of establishing a persistent infection in their host, such that there is continuous virus replication over a long period without killing the host. Another virus-induced demyelination animal model is Semliki Forest virus (SFV) infection of mice [30,31]. The initial immune-mediated demyelination may be due to targeting of SFV-infected oligodendrocytes by cytotoxic T-cells. SFV induces repaired acute demyelination with no relapses.

## Clinical studies supporting a role of virus in MS pathogenesis

The application of modern sophisticated laboratory techniques have led to a growing number of viruses associated with MS albietno such a pathogen has been accepted as the canditate causal agent in MS. In addition, interferon beta, a currently applied treatment in MS patients [32], was originally proposed as being capable of increasing the resistance of host tissues against viral infections. However, no scientific data to date support viral inhibition as one of the underlying mechanisms of action interferon beta in MS.

Several clinical studies have suggested that MS in general as well as episodes of disease exacerbation are associated with concomitant viral or microbial infections[12,33]. Upper respiratory tract infections can trigger acute relapses of MS, resulting in an increase in the risk of clinical exacerbations during the weeks that follow the onset of virus infection[34] Most importantly, when recurrent, these viral infections are associated with neurological progression [35,36].

Many of the studies related to the virus infection in MS are serological and involve the demonstration of increased antibody titers against a particular virus[12]. In addition, in a number of studies, isolation of virus from MS material has also been reported [37]. Antibody levels to various viruses are elevated in MS patients, but it has not been clarified whether this elevation is related to the aetiology of MS or is a concomitant phenomenon. Many viruses have been detected in CNS autopsy tissue from MS patients [38,39]. Throughout the last decades (see table 1 for selected references), among the viral agents related to demyelination were considered the measles virus [40], parainfluenza virus [41], canine distemper [42], Epstein-Barr virus [43], human herpes virus-6 (HHV-6) [44] and retroviruses [45]. It is generally accepted that despite the sensitive methods used, still there is no convincing evidence that viruses are related to MS aetiology, mainly due to controversies among the related studies. HHV-6 in particular, is a typical example of a virus recently tested in details with various assays in order to investigate any relation to MS aetiology[46]. HHV-6 was subjected to detection both in MS patients and healthy controls or patients suffering from other neurological disorders. Patients' material examined was brain tissue [47-49], CSF [50-52], serum/plasma [53-55], and peripheral blood mononuclear cells [53-57] using PCR [47,48,53-57], immunohistochemistry [58-60], or in vitro virus culture assays [58]. Despite such a detailed investigation there is a lack of a conclusive remark on whether HHV-6 is associated to MS[46]. The controversy that is evident throughout the various studies may be attributed either to differences in the sensitivity of the applied methods or the patient selection, different methodology applied, etc. Hence, no matter whether there the viral aetiology of MS is not yet clarified, it should be emphasized that the absence of evidence does not necessary imply the evidence of absence [61].

**Table 1:** Selected studies exploring the relation of multiple sclerosis with human herpes virus-6

**Material used**	**Method followed**	**Relation to MS**	**Year of publication**
Brain tissue	Viral DNA-PCR, immunohistochemistry	Positive	1995[44]
	Viral DNA-PCR	Negative	1996[49]
	Viral DNA-PCR, immunohistochemistry	Positive	1999[47]
	Immunohistochemistry	Positive	2000[58]
	Viral DNA-PCR	Positive	2000[59]
	Viral DNA-PCR, immunohistochemistry	Positive	2003[60]
	Viral DNA-PCR	Uncertain	2003[48]
CSF	Viral DNA-PCR	Negative	1999[50]
	Viral DNA-PCR, antibodies titer	Negative	2000[51]
	Viral DNA-PCR, antibodies titer	Positive	2002[52]
Serum	Viral DNA-PCR	Negative	1999[50]
	Viral DNA-PCR, antibodies titer	Positive	2000[52]
	Viral DNA-PCR	Positive	2000[53]
	Viral DNA-PCR	Positive	2001[54]
	Viral DNA-PCR	Negative	2002[55]
	Viral DNA-PCR, antibodies titer	Positive	2003[56]
Peripheral blood mononuclear cells	Viral DNA-PCR	Negative	2000[51]
	Viral DNA-PCR	Positive	2000[53]
	Viral DNA-PCR	Negative	2000[57]
	Viral DNA-PCR	Positive	2001[54]
	Viral DNA-PCR	Positive	2002[55]
	Viral DNA-PCR	Positive	2003[56]

## Mechanisms of virus-induced CNS autoimmunity

Following a virus infection, there may be two potential options: the virus might reactivate after a long term latency, up to years and lyse oligodendrocytes, or could initiate a rather acute or subacute demyelinating immunopathology. Examples of the first option might be progressive multifocal leucoencephalopathy (PML) through the infection of JC virus (a human papova virus) whereas the later is the case of TMEV encephalomyelitis model, as well as infections with corona viruses, and lenti viruses [62].

Among the major indications for the association of demyelination with viral infection and a destructive host immune response to autoantigens is the case of post-infectious encephalomyelitis, a complication mainly noticed following smallpox vaccination or measles virus and to a lesser extend, varicella and rubella infection. The underlying pathology share similarities with the one induced in EAE [63]. Another example of virus-induced demyelination with concomitant autoimmunity is the one following infection with murine coronavirus in rats. At the acute stage of this animal model demyelination is restricted and related to the infection of glial cells from the virus. However, at later stages, at a time when animals recover from the viral infection, perivascular infiltrates and extented demyelination are present. A transfer EAE was performed by injecting in vitro activated anti-myelin lymphocytes harvested from infected rats at a time that the animals recovered from the initial infection to naive recipients. The resulted EAE was mild with no evidence of demyelination [64]. This finding may indicate that the pre-exposure of the animal to virus may be necessary for the induction of autoimmune demyelination. Similarly, the canine distemper virus (paramyxovirus) – induced demyelination has been reported to be associated with perivascular infiltrations at the late phases following initial infection [65].

The example of TMEV – induced encephalomyelitis is probably the best currently used model of virus-induced immune-mediated demyelination in susceptible mice. TMEV is divided into two subgroups: high-neurovirulent strains, including GDVII and FA, which cause fatal encephalitis, and low-neurovirulent strains, such as DA and BeAn, which cause persistent infection and demyelination in mice [66,67]. The demyelinating phase is preceded by an inflammatory one consisted of macrophages and MHC II – restricted T-cells. However, during the demyelinating phase, cytotoxic and suppressor MHC I – restricted T-cells gradually replace the initial inflammatory subpopulation. In particular, the prevailing opinion is that B- and T-lymphocytes play paradoxical functions in the TMEV-induced CNS disease since they may participate in the virus clearance in CNS cells during the acute phase of the disease and aggravate the demyelinating process in the chronic phase of the disease, thereafter. Therefore, in this model, inflammatory cells are constant components of the underlying demyelinating process [68]. The large number of B- and T-lymphocytes in demyelinating areas suggest that recruitment of these cells into the CNS is an important step in the process of myelin destruction [69-71]. In addition, the response to immunomodulatory or immunosupressent agents [24,72,73] as well as the expression of Ia antigens in glial cells [74], indicate the immune-mediated mechanism of demyelination in this animal model. It was hypothesized that the specificity of primary white matter destruction in the TMEV model depends on immune-sensitised cells, which interact with viral antigen plus MHC antigens on the surfaces of oligodendrocytes or myelin sheaths[75]. The whole underlying pathology shares similarities with the one in MS, except that in the later no viruses are detected in oligodendrocytes as in TMEV encephalomyelitis [76].

**Figure 1:**
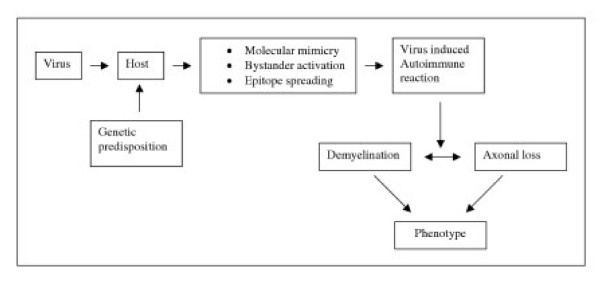
Proposed scheme for virus-mediated autoimmunity in multiple sclerosis.

It was suggested that virus infection may initiate or exacerbate organ-specific autoimmune diseases [77]. There is growing evidence about possible mechanisms by which virus infection can trigger autoimmunity. Among them are: (a) molecular mimicry, (b) bystander activation, and (c) epitope spreading (figure 1).

(a) Molecular mimicry involves the de novo activation of autoreactive T cells due to the cross-reactivity between self epitopes and viral epitopes during a virus infection [78]. Hence, an immune response of the host to a viral epitope will recognize as nonself the crossreacting host epitope even when the virus is no longer present. The concept of molecular mimicry is among the most popular theories about how virus may induce autoimmunity. Accordingly, a molecular mimicry has been reported between anti-TMEV antibody responses in TMEV-infected mice and the myelin component galactocerebroside [67]. In TMEV-induced demyelination, CD4+ T cell responses to myelin epitopes arising via epitope spreading after initial CNS damage approximately 45–60 days post-infection [29]. Interestingly, a molecular mimicry model for initiation of autoimmune demyelination was developed following virus infection with nonpathogenic TMEV which was containing a self myelin epitope such as native or mimic sequences of the immunodominant PLP139-151 epitope. Infection of SJL mice infection with such a virus expressing a self epitope mimic, directly induced autoreactive T cells with pathologic potential in the absence of CFA [79]. The later may be considered as a big advantage of this molecular mimicry model since CFA is a chemical compound imposing artificial inflammatory environment. Alternatively, what is necessary i.e. the CFA, in EAE as a model for autoimmunity in MS, is "provided" in the virus-induced model by the virus per se. The antigen presenting cells (macrophages, dendritic cells, microglia) as well as the capability of the mimic peptide being processed from the native pathogen protein, are two key factors that play important role in the molecular mimicry mechanism during the induction of autoimmunity. In addition, the nature of the innate immune response to the pathogen which determines the immunopathologic potential of the induced cross-reactive T cells, the site(s) of the primary infection in the host and the ability of the pathogen to persist, and finally the potential requirement for multiple infections with the same or different pathogens, are all considered as contributing factors determining the mechanism of molecular mimicry [80].

MBP is among the most important targets in the immunopathogenesis of MS. In addition, the MBP(85–99) peptide is a T-cell target for patients with the HLA-DR2 haplotype while the MBP(88–102) peptide may be a target for patients with other HLA-DR haplotypes. The HLA antigen and the T-cell receptor (TCR) are key factors in the constitution of the trimolecular complex (antigen presenting cell – myelin antigen – T-cell) during lymphocyte activation. Peptide-binding studies determined which MBP peptide residues were important for binding to DR2 and which ones to TCR. These criteria have been applied to generate a minimal molecular mimicry peptide by searching a sequence database for viral and bacterial mimicry peptides of MBP(85–99). Finally, 8 yielded peptides performed biological activity and stimulated MBP(85–99)-specific T-cell clones. These peptides did not show any significant linear homology to MBP(85–99), and they were derived from human pathogens such as EBV, HSV, CMV, influenza virus, and adenovirus [81].

(b) Bystander activation is the nonspecific activation of autoreactive T cells resulting from the direct inflammatory and/or necrotic effects of virus infection on tissue in the target organ [82]. This mechanism requires destruction of specific tissue such as CNS, release of sequestered antigen such as those of myelin and increased local immune inflammation. Lymphocytes would be recruited to the injured CNS and those reactive to the released myelin antigen would in turn be restimulated in the inflammatory response. Consequently, autorreactive lymphocytes would gain access to the target tissue without being directly involved in the initial vira! insult or reactive to viral antigens. Successive targeted viral infections over a lifetime would fulfill the requirement for generation, activation and recruitment of autoimmune lymphocytes. The role of virus in this mechanism is not only to select the tissue, but also to induce a strong inflammatory response [83].

(c) Epitope spreading is characterized by a widening of the immune response from an initiating antigenic epitope to different epitopes on the same molecule (intramolecular spreading) or to a epitopes on a different antigenic molecule (intermolecular spreading). The addition of functional immunogenic myelin epitopes to the original viral epitopes in TMEV infection, represents a classical example of intermolecular epitope spreading [29,84]. In particular, cells responding to the major PLP epitope139-152, isolated from lymph nodes of TMEV infected mice, have the ability to demyelinate organotypic cultures of spinal cord. No similar results were obtained when the cells were stimulated with MBP. These results suggest that in animals infected with TMEV, the spreading of the immune response from TMEV to PLP has functional significance, and is specific[85] T cells specific for a secondary, non-cross-reactive epitope, PLP178-191, have been reported to mediate the primary clinical relapse [86]. This phenomenon has been described in a number of autoimmune diseases, TMEV included [87-90]. More importantly, naive T-cells enter the inflammed CNS and are activated by local antigen presenting cells to initiate epitope spreading [91].

## Axonal degeneration in MS: is there any value for viruses?

Injured axons are common in the lesions of multiple sclerosis, and axonal transection may be the pathologic correlate of the irreversible neurologic impairment in this disease [92]. Axonal degeneration has been identified as the major determinant of irreversible neurological disability in patients with MS. Evidence for the axonal injury – related hypothesis is provided by animal models with primary myelin or axonal pathology, and from pathological or magnetic resonance studies on MS patients [93]. Disruption of axons is observed both in EAE and TMEV models [94].

A correlation between inflammation and axonal loss with neurological disability has been reported in chronic-relapsing EAE. At the acute stage, CNS inflammation, but not axon loss, correlated with the degree of neurological disability. In contrast, fixed neurological impairment in chronic EAE correlated with axon loss. As proposed for MS, these observations imply a causal relationship between inflammation, axon loss, and irreversible neurological disability [95,96]. It has also been demonstrated in TMEV model that demyelination in the spinal cord is followed by a loss of medium to large myelinated fibres. By measuring spinal cord areas, motor-evoked potentials, and motor coordination and balance, axonal loss following demyelination was determined to be associated with electrophysiological abnormalities and correlated strongly with reduced motor coordination and spinal cord atrophy. These findings demonstrate that axonal loss can follow primary, immune-mediated demyelination in the CNS and that the severity of axonal loss correlates almost perfectly with the degree of spinal cord atrophy and neurological deficits [97,98].

Axonal injury begins early at disease onset and correlates with the degree of inflammation within lesions, indicating that inflammatory demyelination influences axon pathology during relapsing-remitting MS (RR-MS) [93]. However, axonal injury exists even in the normal appearing white matter [99] where inflammation may be minimal or absent. In addition, the fact that currently applied immunomodulators and immunosuppresants may hardly reverse or even halt the long term disability and the underlying neurodegeneration, may additionally indicate that there is no absolute relation between the level of inflammation and the extend of axonal degeneration and loss. Moreover, evidence for widespread axonal damage at the earliest clinical stage of MS lessens the validity of the concept that the axonal pathology of MS is the end-stage result of repeated inflammatory events [100].

Interesting information about a possible mechanism under which axonal injury may exist without concomitant demyelination, may emerge from TMEV animal model. The extent and location of axonal injury following infections with both DA and GDVII, TMEV strains, was investigated [101]. In DA virus infection, axonal injury was detected as early as 1 week after infection. The number of damaged axons increased throughout time. During the subclinical phase, 2 and 3 weeks after infection, axonal injury was associated with parenchymal infiltration of microglia and T cells, and viral antigen and damaged axons were present within intact myelin sheaths. However, vigorous inflammatory demyelinating lesions were not seen until the chronic phase (4 weeks after infection). In GDVII virus infection, extensive axonal injury was noted 1 week after infection without association with inflammation, virus, or demyelination. The distribution of injured or damaged axons in both GDVII virus infection and the early phase of DA virus infection corresponded to regions where subsequent demyelination occurred during the chronic phase of DA virus infection. These findings indicate that axonal injury may not follow but rather herald demyelination in some virus models. Somebody may therefore hypothesize that axonal injury noticed during the earliest stages of MS or in the normal appearing white matter, could be attributed to the activation of an as yet unidentified virus. The same or similar virus may further contribute through induced autoimmunity to axonal injury during later stages of the finally established inflammatory demyelinating process.

There are two main animal models currently used in MS research: EAE and TMEV. Both models contributed to a greater understanding of MS and the development of clinical therapies [6]. Although from a first point of view they may represent fanatic supporters of either the autoimmune or the viral hypothesis on MS aetiology, it becomes clear later on that the two models complement each other. Both systems are powerful tools for an in-depth study of the neuroinflammatory mechanisms potentially involved in MS pathophysiology. Analysing therapeutic successes and failures with both models may also help the development of more directed, positive treatments for MS that have fewer negative effects [102].

## References

[B1] Keegan BM, Noseworthy JH (2002). Multiple sclerosis. Annu Rev Med.

[B2] Garren H, Steinman L, Lock C (1998). The specificity of the antibody response in multiple sclerosis. Ann Neurol.

[B3] Martin R, McFarland HF, McFarlin DE (1992). Immunological aspects of demyelinating diseases. Annu Rev Immunol.

[B4] Hafler DA (2004). Multiple sclerosis. J Clin Invest.

[B5] Zamvil SS, Steinman L (1990). The T lymphocyte in experimental allergic encephalomyelitis. Annu Rev Immunol.

[B6] Grigoriadis N, Tselios T, Deraos S, Orologas A, Deraos G, Matsoukas J, Mavromatis I, Milonas I (2005). Animal models of central nervous system immune-mediated diseases: therapeutic interventions with bioactive peptides and mimetics. Curr Med Chem.

[B7] Weiner HL (2004). Multiple sclerosis is an inflammatory T-cell-mediated autoimmune disease. Arch Neurol.

[B8] Barnett MH, Prineas JW (2004). Relapsing and remitting multiple sclerosis: pathology of the newly forming lesion. Ann Neurol.

[B9] Chaudhuri A, Behan PO (2004). Multiple sclerosis is not an autoimmune disease. Arch Neurol.

[B10] Roach ES (2004). Is multiple sclerosis an autoimmune disorder?. Arch Neurol.

[B11] Gilden DH (2002). Multiple sclerosis exacerbations and infection. Lancet Neurol.

[B12] Gilden DH (2005). Infectious causes of multiple sclerosis. Lancet Neurol.

[B13] Lock C, Hermans G, Pedotti R, Brendolan A, Schadt E, Garren H, Langer-Gould A, Strober S, Cannella B, Allard J, Klonowski P, Austin A, Lad N, Kaminski N, Galli SJ, Oksenberg JR, Raine CS, Heller R, Steinman L (2002). Gene-microarray analysis of multiple sclerosis lesions yields new targets validated in autoimmune encephalomyelitis. Nat Med.

[B14] Dyment DA, Ebers GC, Sadovnick AD (2004). Genetics of multiple sclerosis. Lancet Neurol.

[B15] Ibrahim SM, Gold R (2005). Genomics, proteomics, metabolomics: what is in a word for multiple sclerosis?. Curr Opin Neurol.

[B16] Gaudet JP, Hashimoto L, Sadovnick AD, Ebers GC (1995). Is sporadic MS caused by an infection of adolescence and early adulthood? A case-control study of birth order position. Acta Neurol Scand.

[B17] James WH (1988). Further evidence in support of the hypothesis that one cause of multiple sclerosis is childhood infection. Neuroepidemiology.

[B18] Kurland LT (1994). The evolution of multiple sclerosis epidemiology. Ann Neurol.

[B19] Kurtzke JF (1993). Epidemiologic evidence for multiple sclerosis as an infection. Clin Microbiol Rev.

[B20] Sotgiu S, Pugliatti M, Fois ML, Arru G, Sanna A, Sotgiu MA, Rosati G (2004). Genes, environment, and susceptibility to multiple sclerosis. Neurobiol Dis.

[B21] Poser CM (1986). Pathogenesis of multiple sclerosis. A critical reappraisal. Acta Neuropathol (Berl).

[B22] Allen I, Brankin B (1993). Pathogenesis of multiple sclerosis--the immune diathesis and the role of viruses. J Neuropathol Exp Neurol.

[B23] Fazakerley JK, Buchmeier MJ (1993). Pathogenesis of virus-induced demyelination. Adv Virus Res.

[B24] Lipton HL, Dal Canto MC (1976). Theiler's virus-induced demyelination: prevention by immunosuppression. Science.

[B25] Schlitt BP, Felrice M, Jelachich ML, Lipton HL (2003). Apoptotic cells, including macrophages, are prominent in Theiler's virus-induced inflammatory, demyelinating lesions. J Virol.

[B26] Weiner LP (1973). Pathogenesis of demyelination induced by a mouse hepatitis. Arch Neurol.

[B27] Lampert PW, Sims JK, Kniazeff AJ (1973). Mechanism of demyelination in JHM virus encephalomyelitis. Electron microscopic studies. Acta Neuropathol (Berl).

[B28] Powell HC, Lampert PW (1975). Oligodendrocytes and their myelin-plasma membrane connections in JHM mouse hepatitis virus encephalomyelitis. Lab Invest.

[B29] Miller SD, Vanderlugt CL, Begolka WS, Pao W, Yauch RL, Neville KL, Katz-Levy Y, Carrizosa A, Kim BS (1997). Persistent infection with Theiler's virus leads to CNS autoimmunity via epitope spreading. Nat Med.

[B30] Atkins GJ, Sheahan BJ, Dimmock NJ (1985). Semliki Forest virus infection of mice: a model for genetic and molecular analysis of viral pathogenicity. J Gen Virol.

[B31] Atkins GJ, Sheahan BJ, Liljestrom P (1999). The molecular pathogenesis of Semliki Forest virus: a model virus made useful?. J Gen Virol.

[B32] Grigoriadis N (2002). Interferon beta treatment in relapsing-remitting multiple sclerosis. A review. Clin Neurol Neurosurg.

[B33] Buljevac D, Flach HZ, Hop WC, Hijdra D, Laman JD, Savelkoul HF, van Der Meche FG, van Doorn PA, Hintzen RQ (2002). Prospective study on the relationship between infections and multiple sclerosis exacerbations. Brain.

[B34] Kriesel JD, Sibley WA (2005). The case for rhinoviruses in the pathogenesis of multiple sclerosis. Mult Scler.

[B35] Panitch HS (1994). Influence of infection on exacerbations of multiple sclerosis. Ann Neurol.

[B36] Edwards S, Zvartau M, Clarke H, Irving W, Blumhardt LD (1998). Clinical relapses and disease activity on magnetic resonance imaging associated with viral upper respiratory tract infections in multiple sclerosis. J Neurol Neurosurg Psychiatry.

[B37] Clark DA (1999). Human herpesvirus 6 and multiple sclerosis. Herpes.

[B38] Haahr S, Sommerlund M, Christensen T, Jensen AW, Hansen HJ, Moller-Larsen A (1994). A putative new retrovirus associated with multiple sclerosis and the possible involvement of Epstein-Barr virus in this disease. Ann N Y Acad Sci.

[B39] Christensen T, Dissing Sorensen P, Riemann H, Hansen HJ, Moller-Larsen A (1998). Expression of sequence variants of endogenous retrovirus RGH in particle form in multiple sclerosis. Lancet.

[B40] Field EJ, Cowshall S, Narang HK, Bell TM (1972). Viruses in multiple sclerosis?. Lancet.

[B41] ter Meulen V, Koprowski H, Iwasaki Y, Kackell YM, Muller D (1972). Fusion of cultured multiple-sclerosis brain cells with indicator cells: presence of nucleocapsids and virions and isolation of parainfluenza-type virus. Lancet.

[B42] Cook SD, Dowling PC (1977). A possible association between house pets and multiple sclerosis. Lancet.

[B43] Ascherio A, Munger KL, Lennette ET, Spiegelman D, Hernan MA, Olek MJ, Hankinson SE, Hunter DJ (2001). Epstein-Barr virus antibodies and risk of multiple sclerosis: a prospective study. Jama.

[B44] Challoner PB, Smith KT, Parker JD, MacLeod DL, Coulter SN, Rose TM, Schultz ER, Bennett JL, Garber RL, Chang M (1995). Plaque-associated expression of human herpesvirus 6 in multiple sclerosis. Proc Natl Acad Sci U S A.

[B45] Perron H, Garson JA, Bedin F, Beseme F, Paranhos-Baccala G, Komurian-Pradel F, Mallet F, Tuke PW, Voisset C, Blond JL, Lalande B, Seigneurin JM, Mandrand B (1997). Molecular identification of a novel retrovirus repeatedly isolated from patients with multiple sclerosis. The Collaborative Research Group on Multiple Sclerosis. Proc Natl Acad Sci U S A.

[B46] Clark D (2004). Human herpesvirus type 6 and multiple sclerosis. Herpes.

[B47] Friedman JE, Lyons MJ, Cu G, Ablashl DV, Whitman JE, Edgar M, Koskiniemi M, Vaheri A, Zabriskie JB (1999). The association of the human herpesvirus-6 and MS. Mult Scler.

[B48] Cermelli C, Berti R, Soldan SS, Mayne M, D'Ambrosia J M, Ludwin SK, Jacobson S (2003). High frequency of human herpesvirus 6 DNA in multiple sclerosis plaques isolated by laser microdissection. J Infect Dis.

[B49] Sanders VJ, Felisan S, Waddell A, Tourtellotte WW (1996). Detection of herpesviridae in postmortem multiple sclerosis brain tissue and controls by polymerase chain reaction. J Neurovirol.

[B50] Mirandola P, Stefan A, Brambilla E, Campadelli-Fiume G, Grimaldi LM (1999). Absence of human herpesvirus 6 and 7 from spinal fluid and serum of multiple sclerosis patients. Neurology.

[B51] Taus C, Pucci E, Cartechini E, Fie A, Giuliani G, Clementi M, Menzo S (2000). Absence of HHV-6 and HHV-7 in cerebrospinal fluid in relapsing-remitting multiple sclerosis. Acta Neurol Scand.

[B52] Tejada-Simon MV, Zang YC, Hong J, Rivera VM, Killian JM, Zhang JZ (2002). Detection of viral DNA and immune responses to the human herpesvirus 6 101-kilodalton virion protein in patients with multiple sclerosis and in controls. J Virol.

[B53] Akhyani N, Berti R, Brennan MB, Soldan SS, Eaton JM, McFarland HF, Jacobson S (2000). Tissue distribution and variant characterization of human herpesvirus (HHV)-6: increased prevalence of HHV-6A in patients with multiple sclerosis. J Infect Dis.

[B54] Tomsone V, Logina I, Millers A, Chapenko S, Kozireva S, Murovska M (2001). Association of human herpesvirus 6 and human herpesvirus 7 with demyelinating diseases of the nervous system. J Neurovirol.

[B55] Alvarez-Lafuente R, Martin-Estefania C, de Las Heras V, Castrillo C, Picazo JJ, Varela de Seijas E, Gonzalez RA (2002). Active human herpesvirus 6 infection in patients with multiple sclerosis. Arch Neurol.

[B56] Chapenko S, Millers A, Nora Z, Logina I, Kukaine R, Murovska M (2003). Correlation between HHV-6 reactivation and multiple sclerosis disease activity. J Med Virol.

[B57] Ferrante P, Mancuso R, Pagani E, Guerini FR, Calvo MG, Saresella M, Speciale L, Caputo D (2000). Molecular evidences for a role of HSV-1 in multiple sclerosis clinical acute attack. J Neurovirol.

[B58] Knox KK, Brewer JH, Henry JM, Harrington DJ, Carrigan DR (2000). Human herpesvirus 6 and multiple sclerosis: systemic active infections in patients with early disease. Clin Infect Dis.

[B59] Blumberg BM, Mock DJ, Powers JM, Ito M, Assouline JG, Baker JV, Chen B, Goodman AD (2000). The HHV6 paradox: ubiquitous commensal or insidious pathogen? A two-step in situ PCR approach. J Clin Virol.

[B60] Goodman AD, Mock DJ, Powers JM, Baker JV, Blumberg BM (2003). Human herpesvirus 6 genome and antigen in acute multiple sclerosis lesions. J Infect Dis.

[B61] Fazakerley JK, Walker R (2003). Virus demyelination. J Neurovirol.

[B62] Buchmeier MJ, Lane TE (1999). Viral-induced neurodegenerative disease. Curr Opin Microbiol.

[B63] Lampert PW (1978). Autoimmune and virus-induced demyelinating diseases. A review. Am J Pathol.

[B64] Watanabe R, Wege H, ter Meulen V (1983). Adoptive transfer of EAE-like lesions from rats with coronavirus-induced demyelinating encephalomyelitis. Nature.

[B65] Krakowka S, McCullough B, Koestner A, Olsen R (1973). Myelin-specific autoantibodies associated with central nervous system demyelination in canine distemper virus infection. Infect Immun.

[B66] Tsunoda IFRS, R Ahmed IC (1999). Theiler's murine encephalomyelitis virus.. Pesistent viral infections.

[B67] Tsunoda I, Fujinami RS (1996). Two models for multiple sclerosis: experimental allergic encephalomyelitis and Theiler's murine encephalomyelitis virus. J Neuropathol Exp Neurol.

[B68] Dal Canto MC, Lipton HL (1975). Primary demyelination in Theiler's virus infection. An ultrastructural study. Lab Invest.

[B69] Yauch RL, Kim BS (1994). A predominant viral epitope recognized by T cells from the periphery and demyelinating lesions of SJL/J mice infected with Theiler's virus is located within VP1(233-244). J Immunol.

[B70] Kurtz CI, Sun XM, Fujinami RS (1995). B-lymphocyte requirement for vaccine-mediated protection from Theiler's murine encephalomyelitis virus-induced central nervous system disease. J Virol.

[B71] Rodriguez M, Pavelko KD, Njenga MK, Logan WC, Wettstein PJ (1996). The balance between persistent virus infection and immune cells determines demyelination. J Immunol.

[B72] Roos RP, Firestone S, Wollmann R, Variakojis D, Arnason BG (1982). The effect of short-term and chronic immunosuppression on Theiler's virus demyelination. J Neuroimmunol.

[B73] Rodriguez M, Lafuse WP, Leibowitz J, David CS (1986). Partial suppression of Theiler's virus-induced demyelination in vivo by administration of monoclonal antibodies to immune-response gene products (Ia antigens). Neurology.

[B74] Rodriguez M, Pierce ML, Howie EA (1987). Immune response gene products (Ia antigens) on glial and endothelial cells in virus-induced demyelination. J Immunol.

[B75] Rodriguez M, Oleszak E, Leibowitz J (1987). Theiler's murine encephalomyelitis: a model of demyelination and persistence of virus. Crit Rev Immunol.

[B76] Rodriguez M, Leibowitz JL, Lampert PW (1983). Persistent infection of oligodendrocytes in Theiler's virus-induced encephalomyelitis. Ann Neurol.

[B77] Olson JK, Croxford JL, Miller SD (2001). Virus-induced autoimmunity: potential role of viruses in initiation, perpetuation, and progression of T-cell-mediated autoimmune disease. Viral Immunol.

[B78] Fujinami RS, Oldstone MB (1985). Amino acid homology between the encephalitogenic site of myelin basic protein and virus: mechanism for autoimmunity. Science.

[B79] Olson JK, Croxford JL, Calenoff MA, Dal Canto MC, Miller SD (2001). A virus-induced molecular mimicry model of multiple sclerosis. J Clin Invest.

[B80] Olson JK, Ludovic Croxford J, Miller SD (2004). Innate and adaptive immune requirements for induction of autoimmune demyelinating disease by molecular mimicry. Mol Immunol.

[B81] Wucherpfennig K, WC R (1997). T cell mediated autoimmunity in multiple sclerosis. Molecular biology of multiple sclerosis.

[B82] Horwitz MS, Bradley LM, Harbertson J, Krahl T, Lee J, Sarvetnick N (1998). Diabetes induced by Coxsackie virus: initiation by bystander damage and not molecular mimicry. Nat Med.

[B83] Horwitz MS, Sarvetnick N (1999). Viruses, host responses, and autoimmunity. Immunol Rev.

[B84] Vanderlugt CL, Miller SD (2002). Epitope spreading in immune-mediated diseases: implications for immunotherapy. Nat Rev Immunol.

[B85] Dal Canto MC, Calenoff MA, Miller SD, Vanderlugt CL (2000). Lymphocytes from mice chronically infected with Theiler's murine encephalomyelitis virus produce demyelination of organotypic cultures after stimulation with the major encephalitogenic epitope of myelin proteolipid protein. Epitope spreading in TMEV infection has functional activity. J Neuroimmunol.

[B86] McRae BL, Vanderlugt CL, Dal Canto MC, Miller SD (1995). Functional evidence for epitope spreading in the relapsing pathology of experimental autoimmune encephalomyelitis. J Exp Med.

[B87] Katz-Levy Y, Neville KL, Padilla J, Rahbe S, Begolka WS, Girvin AM, Olson JK, Vanderlugt CL, Miller SD (2000). Temporal development of autoreactive Th1 responses and endogenous presentation of self myelin epitopes by central nervous system-resident APCs in Theiler's virus-infected mice. J Immunol.

[B88] Miller SD, Katz-Levy Y, Neville KL, Vanderlugt CL (2001). Virus-induced autoimmunity: epitope spreading to myelin autoepitopes in Theiler's virus infection of the central nervous system. Adv Virus Res.

[B89] Vanderlugt CL, Begolka WS, Neville KL, Katz-Levy Y, Howard LM, Eagar TN, Bluestone JA, Miller SD (1998). The functional significance of epitope spreading and its regulation by co-stimulatory molecules. Immunol Rev.

[B90] Tuohy VK, Yu M, Yin L, Kawczak JA, Kinkel RP (1999). Spontaneous regression of primary autoreactivity during chronic progression of experimental autoimmune encephalomyelitis and multiple sclerosis. J Exp Med.

[B91] McMahon EJ, Bailey SL, Castenada CV, Waldner H, Miller SD (2005). Epitope spreading initiates in the CNS in two mouse models of multiple sclerosis. Nat Med.

[B92] Trapp BD, Peterson J, Ransohoff RM, Rudick R, Mork S, Bo L (1998). Axonal transection in the lesions of multiple sclerosis. N Engl J Med.

[B93] Bjartmar C, Wujek JR, Trapp BD (2003). Axonal loss in the pathology of MS: consequences for understanding the progressive phase of the disease. J Neurol Sci.

[B94] Bjartmar C, Yin X, Trapp BD (1999). Axonal pathology in myelin disorders. J Neurocytol.

[B95] Wujek JR, Bjartmar C, Richer E, Ransohoff RM, Yu M, Tuohy VK, Trapp BD (2002). Axon loss in the spinal cord determines permanent neurological disability in an animal model of multiple sclerosis. J Neuropathol Exp Neurol.

[B96] Grigoriadis N, Ben-Hur T, Karussis D, Milonas I (2004). Axonal damage in multiple sclerosis: a complex issue in a complex disease. Clin Neurol Neurosurg.

[B97] McGavern DB, Murray PD, Rivera-Quinones C, Schmelzer JD, Low PA, Rodriguez M (2000). Axonal loss results in spinal cord atrophy, electrophysiological abnormalities and neurological deficits following demyelination in a chronic inflammatory model of multiple sclerosis. Brain.

[B98] Ure D, Rodriguez M (2000). Extensive injury of descending neurons demonstrated by retrograde labeling in a virus-induced murine model of chronic inflammatory demyelination. J Neuropathol Exp Neurol.

[B99] Evangelou N, Esiri MM, Smith S, Palace J, Matthews PM (2000). Quantitative pathological evidence for axonal loss in normal appearing white matter in multiple sclerosis. Ann Neurol.

[B100] Filippi M, Bozzali M, Rovaris M, Gonen O, Kesavadas C, Ghezzi A, Martinelli V, Grossman RI, Scotti G, Comi G, Falini A (2003). Evidence for widespread axonal damage at the earliest clinical stage of multiple sclerosis. Brain.

[B101] Tsunoda I, Kuang LQ, Libbey JE, Fujinami RS (2003). Axonal injury heralds virus-induced demyelination. Am J Pathol.

[B102] Nelson AL, Bieber AJ, Rodriguez M (2004). Contrasting murine models of MS. Int MS J.

